# Youth educational mobility and the rural family in China

**DOI:** 10.1177/00345237231216309

**Published:** 2023-11-24

**Authors:** Haoyang Zhang, Li-Chung Hu, Emily Hannum

**Affiliations:** Department of Sociology and Criminology, 8082 Pennsylvania State University, University Park, PA, USA; Department of Sociology, 34913 National Chengchi University, Taipei City, Taiwan; Department of Sociology and Population Studies Center, 6572 University of Pennsylvania, Philadelphia, PA, USA

**Keywords:** Educational mobility, second demographic transition theory, rural education, rural youth, family, individualization

## Abstract

Urban-rural economic opportunity gaps drive rural youth to seek economic stability by migrating away from home and family. The links between educational attainment and economic outcomes for rural youth are well studied in China and elsewhere, but the implications of educational mobility for rural family relationships remain less understood. Extending tenets of second demographic transition theory, we posit that education sets the stage for “individualization:” geographic mobility for urban work distant from rural family networks. Educational mobility may thus set conditions for upending traditional family co-residence patterns, direct-support relationships, and family-gender attitudes. In this paper, we consider first whether educational advancement is associated with urban economic mobility for rural youth in young adulthood, and then ask whether education is linked to disruption of rural family relations. Specifically, using the case of children growing up in rural northwest China, we estimate relationships between secondary and tertiary educational attainment and (1) economic stability, (2) intergenerational geographic separation and material exchange, and (3) traditional family and gender role attitudes, after adjusting for potential confounders. Results show that for both men and women, education is associated with greater economic stability in young adulthood, across several measures, and with an erosion of adherence to traditional family and gender attitudes. Moreover, for men, education correlates to less family proximity and more material exchange.

## Introduction

Education is commonly viewed as a key pathway to economic security for youth, especially for those from impoverished rural communities. Both young people and their parents often firmly believe that education can enhance their family’s social and economic status (Basnet et al., 2021). At the same time, vast disparities in educational resources and living standards between rural and urban areas contribute to high levels of inequality in many low- and middle-income countries (Lagakos, 2020). Due to urban-rural divides, youth in rural areas often face limited prospects, compared to their urban counterparts (Suttie, 2019: 1–2).

A noteworthy example of these phenomena can be seen in China, where rapid development and urbanization have raised access to education and the standard of living while also increasing the economic and educational opportunity gaps that rural children face compared to wealthier urban counterparts (Kong et al., 2021; Roberts and Hannum, 2018). In urban areas, the quality of education has long surpassed what is available in rural China: urban schools offer more resources, better infrastructure, and more qualified teachers (for example, see Zhao and Glewwe, 2010).^1^ A legacy of educational access gaps and outmigration have left stark educational attainment differences between the adult populations in rural and urban areas: according to 2015 intercensal survey data, only 14% of the rural labor force has a high school (or higher) education, compared with 42% of the urban labor force (National Bureau of Statistics of China, 2017). And while education without doubt presents a path to economic security for some rural students, the estimated returns to education in rural areas of China are disappointingly low, notwithstanding significant government investment in rural education in recent decades (Liu et al., 2020). Importantly, when economic and occupational opportunities are made possible via education, these opportunities have tended to be far from home and family for rural youth. Educational mobility has become tethered to the notion of departure to an urban future for many youth and their parents in rural China (Kong, 2010; 2015).

The links between education and economic outcomes are well studied empirically in rural China and elsewhere, but implications of educational mobility for rural family ties and processes are less well theorized and documented. A set of demographic theories offers a framework for thinking about these implications. Education—particularly women’s education—received much attention in classic theories of the “first” demographic transition, for its role in lowering fertility and child mortality (Goujon, 2008), with impacts occurring not only through improved maternal literacy but through economic empowerment, ties to non-agricultural employment that disincentivized large families, and connections to more urban and consumption-based lifestyles.

More recently, theories of the so-called “second demographic transition” described by Lesthaeghe and van de Kaa (Lesthaeghe, 2014, 2020; original source cited as Lesthaeghe and Van de Kaa, 1986, in Dutch) considered population trends that include postponed family formation and the rise of cohabitation (and sometimes non-marital fertility) around the world. Second demographic transition theory (hereafter SDT) emphasizes population shifts towards more individualistic values, increased female empowerment, secularization, and a move away from traditional family norms. With the exception that there is a continued aversion to non-marital childbearing, trends consistent with second demographic transition have also emerged in China (Yu and Xie, 2022). Education can be viewed in this framework as a catalyst for ideational change, individualization, women’s empowerment, and individual economic mobility.^2^

What does SDT imply about the impact of educational mobility on relations with families of origin in rural China? Much of the existing SDT literature in China has focused on shifts in family formation—including marriage timing, cohabitation, and non-marital fertility, with some attention to how these patterns vary by education (Yu and Xie, 2015; 2022). Here, we suggest that SDT also implies certain disruptions to rural family intergenerational relations associated with new educational opportunities: education is associated with distant economic opportunities and an urban lifestyle, and thus may inadvertently erode traditional family co-residence patterns, direct-support relationships, and family-gender attitudes. We focus on how the notion of individualization in SDT may apply to rural family relations—how education sets conditions for diminishing adherence to specific traditional family practices and beliefs.

To pursue this argument, we focus on the case of rural youth growing up in one of China’s poorest provinces. Our analysis proceeds in two stages. We first consider whether education in this setting is associated with economic security in young adulthood, and then ask whether educational opportunity is linked to erosion of traditional rural family relationships and beliefs. Specifically, drawing on a 15-year longitudinal survey of rural youth, we estimate relationships between secondary and tertiary educational attainment and (1) markers of economic stability and urban residency in young adulthood, (2) a shift toward geographic separation and intergenerational material exchange, and (3) erosion of traditional family and gender role attitudes, after adjusting for many potential confounders. Because traditional patrilocal marriage patterns mean that geographic separation from natal families in adulthood has long been more common for young rural women than men, we consider results separately for men and women.

Our results show that education is associated with urban-based economic mobility in young adulthood, for both men and women, and that it is associated with a disruption of certain traditional family and gender attitudes for both groups. However, in part because young women have not been expected to continue to live with natal families after marriage, a tradeoff between family proximity and material exchange is observed primarily for young men. We discuss the implications of these patterns in our conclusion.

## Framework

Second demographic transition theory argues that individualization and other social trends support demographic shifts such as postponement of marriage and childbearing, the decline of fertility and marriage rates, increased cohabitation, rising divorce rates, and sub-replacement fertility levels (Lesthaeghe, 2010). A shift towards self-fulfillment over traditional family obligations is an essential element of SDT (Lesthaeghe, 2010). Central to this theory is the phenomenon of urbanization. Urban life is considered in SDT to be conducive to new forms of family formation and interaction that displace the old. In many countries, urban areas are viewed as hubs of economic opportunity, as they feature a concentration of diverse and quality resources that are not commonly found in rural settings, in part due to dense concentration of industries and businesses (Glaeser and Gottlieb, 2009). Urban areas tend to house a diverse array of job markets and, therefore, provide a broad spectrum of potential careers for educated individuals moving from rural areas. For example, according to the United States Department of Agriculture (USDA), individuals migrating from rural to urban regions in the United States generally witness substantial increases in earnings (McGranahan et al., 2010). Education-facilitated outmigration, therefore, can be a critical driver of upward social mobility among rural youth.^3^

As urban areas expand and become centers of economic, educational, and social opportunities, rural areas often experience a drain of their youth populations, who seek better prospects in urban areas (Brown and Schafft, 2011; Sano et al., 2020). Yet, rural-to-urban migrants often face barriers such as cultural adjustments, unfamiliar environments, and the unpredictability of urban returns on their educational investments. Urban centers might expose rural youth to challenges like culture shock, identity crises, and the potential erosion of traditional values (Portes and Zhou, 1993). Additionally, transitioning from a rural to an urban area can lead to social isolation. Youth relocating to urban areas for education or employment frequently grapple with loneliness due to the loss of their support network (Lu, 2010). This shift has knock-on effects on the structure of rural families and the nature of intergenerational relationships (Albertini et al., 2019; Baykara-Krumme and Fokkema, 2019; Karpinska and Dykstra, 2019). On the economic front, urban opportunities might not always be accessible to rural-origin migrants. The urban job market often favors individuals possessing skills and networks nurtured in urban contexts, leaving rural migrants in a disadvantaged position (Sherman and Sage, 2011). Furthermore, the cost of living in urban regions is typically higher, posing a financial burden on rural youth, particularly if they are low-wage earners. The elevated cost of living in urban areas can counterbalance the advantages gained from increased earnings and impose new forms of stress (Chen and Rosenthal, 2008), alongside isolation.  

To the extent that economic opportunities are perceived to exist primarily in cities, youth educational mobility has seemingly-unavoidable implications for some kinds of social relationships that have been prevalent in rural areas in many countries. In many rural communities, intergenerational relationships that are often facilitated through co-residence serve as a primary source of support (De Valk and Bordone, 2019). Co-residence or geographical proximity is crucial for certain types of personal care requiring physical presence (Giles and Mu, 2007). Yet, as youth outmigration increases, the means of intergenerational support may also diversify. Remittances from migrants and the use of communication technologies introduce alternate ways for generations to support each other materially and socially (Zimmer and Kwong, 2003). While numerous studies have delved into intergenerational relations, their findings remain inconclusive regarding the implications of migration for intergenerational bonds (Albertini et al., 2019). For instance, transnational migrant families in Germany, who have migrated from Turkey, exhibit weaker intergenerational relationships than their counterparts who remain in Turkey (Baykara-Krumme and Fokkema, 2019). In contrast, geographic separation resulting from migration does not appear to disrupt intergenerational relationships or obligations in Polish transnational families (Karpinska and Dykstra, 2019). 

In short, urbanization and individualization are important concepts in SDT. From the perspective of rural youth, educational mobility is an essential thread in the urbanization process. In principle, urbanization plays a pivotal role in propelling the instrumental value of education. Conversely, education may act as a catalyst, accelerating and reinforcing the process of urbanization and exerting transformative pressures on the rural family.

## The case of China

China is experiencing many patterns consistent with second demographic transition theory (Yu and Xie, 2022). Rapid industrialization and urbanization since the 1990s have prompted an unprecedented rise of internal migration (Liang and Song, 2016). Internal migration in China typically involves moving from underdeveloped regions, such as rural areas, to resource-rich ones, such as urban areas. Youth living in rural communities in China tend to experience dense, often kin-based social networks (for a discussion, see Shen and Hannum, 2023: 727); such networks are likely to be much more sparse for youth migrants into cities. Moreover, consistent with notions of individualization, for rural youth in China, the appeal of urban life has been linked to a desire for self-cultivation (自我发展, *ziwofazhan*), as well as economic opportunity (Chiang et al., 2015).

China has seen educational expansion, even more rapidly for women than for men, though male-favoring disparities in educational opportunities may remain a significant concern for the poorest families and in poor rural regions (Hannum et al., 2009). Moreover, massive population migration in China has not only changed economic patterns in rural communities but also dramatically reshaped Chinese family dynamics and traditional gender roles. Traditionally, Chinese families have been predominantly patrilocal and patrilineal, centering on male descent lines (Chen et al., 2000; Zhang, 2004). Sons were expected to be the main providers of elder care, while married daughters, considered part of their husbands’ families, were expected to care for their in-laws rather than their own parents (Lee and Xiao, 1998). Within a marital context, conventional Chinese gender roles positioned the husband as responsible for economic and productive activities, while the wife played a complementary role—supporting her husband's productivity and managing domestic responsibilities like child-rearing (Hu and Scott, 2016). However, large-scale rural-to-urban migration has begun to challenge these traditional expectations around family and gender roles. For example, as increasing numbers of young men migrate for labor and live apart from their families, daughters remaining at home play an increasingly critical role in supporting their families (Liu, 2014).^4^

The relationship between education, outmigration, and ideological shifts concerning traditional family and gender roles may also correspond to changes in family dynamics. Chinese culture, historically valuing intergenerational care, draws from the Confucian concept of filial piety (孝, *xiao*), which prescribes obedience to older generations and is often viewed as implying a duty to care for the elderly at home (Tu, 2016). In this cultural context, home-based elder care is perceived as a moral obligation. However, significant population movements have diminished familial in-person contact and transformed the nature of intergenerational relationships. Younger generations migrating to urban areas often find themselves geographically distanced from their native families who remain in rural regions (Liu, 2017). This geographic separation for educational advancement presents a “trade-off between instrumental and financial support” where those living with their parents are capable of providing physical support, whereas those living apart from home are more likely to contribute financially than physically (Zimmer and Kwong, 2003: 34).

In short, family dynamics and societal norms are continually evolving, driven by numerous factors. Education stands out as a powerful agent of change. Serving as both a tool for empowerment and a bridge between tradition and modernity, education not only carries the potential to equip individuals with skills for economic security but also reshapes established family norms and dynamics. In the analyses that follow, we investigate the associations among education, transition to urban life and economic security, and individualization. We hypothesize that more education will correlate to enhanced financial security and a greater likelihood of physical distance from one's natal family. Furthermore, those with higher education may lean more towards material intergenerational support over interpersonal relationships. Such individuals are also expected to question traditional family values, especially concerning co-residency and son preference. While we are not able to isolate causal effects of education with our design, we are able to estimate a series of relationships that are highly suggestive of the potential role of educational mobility as a bridge to urbanization and alternative forms of family interaction—key elements in the individualization processes associated with SDT.

## Method

### Study site and sample

We focus on the case of children growing up in rural Gansu Province, which is located in China’s arid Northwest and is one of China’s poorest provinces. The province is long and narrow, stretching across a desert, mountainous and hilly areas, and vast grasslands. We implemented a study in rural Gansu villages—Gansu Survey of Children and Families (GSCF)—to illuminate children’s educational experiences and attainment, development, welfare, and economic mobility. The baseline wave of the study took place in 2000, and the most recent wave of data collection took place in 2015. This project consisted of 2000 children who lived in 100 rural villages located in 20 counties of Gansu Province. Drawing on a multi-stage cluster sample of rural households with children in the target age range, the sampled children were first interviewed in the year 2000 and last interviewed in early adulthood in the year 2015. The questionnaires were administered at schools and in homes to children, teachers, school principals, mothers, and household heads. Our sample consists of children who were aged 9 to 12 in the year 2000 (GSCF-1) and in their mid-20s in 2015. In 2015, 1613 now-adult children of the initial sample were followed up. For this paper, we dropped cases that were missing on outcomes or independent variables.

### Measuring youth economic outcomes and stressors on the rural family

The outcomes we consider in this study were grouped into three categories—economic stability, family separation and relations, and family attitudes. For economic stability, we used *non-rural hukou*, *current* *employment status*, having a *labor contract*,^5^ having an *old age pension,* and having a *housing provident fund* to capture different aspects of economic stability in rural China. These five measures are binary variables.

Though policies are evolving, hukou status has long profoundly affected individual life chances in China. As a form of identity formalized by the government, the household registration or hukou system has important implications for individuals in China (Roberts and Hannum, 2018). An urban hukou grants various social benefits, and rural residency, in particular, limits rural populations’ ability to access social services, education, and urban welfare (Chen, 2020; Roberts and Hannum, 2018). For this reason, large numbers of rural individuals out-migrated to urban areas in order to seek better educational and economic resources. Despite the fact that educational policies have evolved to accommodate this large-scale internal migration, rural hukou has restricted the kind of education a student can receive (Roberts and Hannum, 2018). It is difficult to convert from rural to urban hukou, but education provides one possible avenue for rural youth to cross the rural-urban boundary (Wu and Treiman, 2004). For the variable *non-rural hukou*, if respondents have a non-rural hukou, the variable is coded as 1. Otherwise, it is coded as 0.

Current *employment status* captures whether respondents were currently employed or working as farmers, self-employed and working for a family business. Prior research suggested that off-farm jobs can significantly contribute to poverty alleviation in rural China (Li et al., 2021). Thus, we considered being employed as an important attribute of economic stability. The *current employment status* variable was coded as 1 for respondents who were currently employed, and 0 for unemployed.

Furthermore, we measured whether respondents have *labor contracts.* Not all workers sign formal contracts with their employers, and this is especially true for migrant workers. As a result, employees are restrained from accessing job-related benefits (Gao et al., 2012). Therefore, whether employees sign labor contracts with their employers provides a direct and intuitive measurement of economic stability. We also included three common job-related benefits—*old age pension*, *medical insurance,* and access to the *housing provident fund*^6^—as dependent variables for supplementary measurements of economic stability, to further discern which types of job-related benefits respondents enjoy.

*Geographic separation* was measured by where respondents currently reside, including living at home, living in the same village, living in the same township but a different village, living in the same county but a different township, living in the same province but a different county, and living in other provinces. These categories approximately reflect how far respondents are geographically separated from their parents. For simplicity, we used a dichotomous variable to indicate whether respondents currently live within or outside the county. Respondents who currently live in the same province but in a different county or in other provinces were regarded as “outside the county”, and all other categories were categorized as “within the county.”^7^

We used *material exchange* to capture a form of family relationship that may be particularly important when labor migration precludes co-residence and direct support. Out-migration to work among young adults has become prevalent, such that sending money home is a common phenomenon. Literature regarding remittances suggests that strong family ties or obligations are dominant motivators for sending remittances among rural-to-urban migrants (Akay et al., 2014; Cai, 2003). Thus, whether respondents send money home provides an indirect measurement of family relationships in this context. Importantly, existing literature shows that intergenerational financial transfers tend to be bidirectional (Cong and Silverstein, 2011). Not only do parents receive money from children; they also may invest in their children when they expect that investment to benefit the child or family. We constructed material exchange into five categories, contrasting types of material exchange to a base category of co-residency (i.e., living together with parents; in the survey, those living together with parents did not report material exchange). The categories are, separate and giving money to parents only, separate and receiving money from parents only, separate and both giving money to and receiving money from parents, and separate and no material exchange.

Family attitudes encompass both intergenerational attitudes and gender role attitudes. Historically, the Chinese family in most parts of the country has been characterized as patrilocal and patrilineal (Thornton and Lin, 1994). Chinese parents tend to expect children—especially sons— to co-reside with them and take care of them in their old age (Hannum et al., 2009; Pei and Cong, 2020). To assess the association between intergenerational attitudes and education, we used two 5-point Likert scale measures ranging from 1 (strongly disagree) to 5 (strongly agree): 1. *ideal to live separately:* If economic and health conditions of parents are fine, it is ideal to live separately from parents; and 2. *son preference:* In the ideal situation, couples should have a son. These two items capture two core aspects of intergenerational attitudes, i.e., co-residency and offspring sex preference. Additionally, another two 5-point Likert scale items were utilized to measure agreement with gender role attitudes: 1. *gendered division of labor*: Husband focuses on outside work while wife focuses on the family; and 2. *gendered childcare responsibilities:* Mothers should do the lion’s share of childcare. These two items are widely used for measuring gender role attitudes in survey studies (Davis and Greenstein, 2009). For regression models, the variables s*on preference, gendered division of labor,* and *gendered childcare responsibilities *were reverse-coded to generate *less* s*on preference, less gendered division of labor*, and *less* g*endered childcare responsibilities, *so that higher scores indicated stronger non-traditional or egalitarian attitudes toward family or gender roles. All of the dependent variables were measured in the 2015 survey and were reported by the sample children, except material exchange. Material exchange was reported in the household questionnaire.

### Measuring youth educational attainment

In the 2015 survey, respondents were asked about their highest degree of education attained. Based on their responses, we generated a categorical measure of *education* measuring the highest degree attained, including middle school and less, secondary education, and college and above.

### Adjusting for marital status and background characteristics

We also included sample children’s *marital status* in the analysis. Existing literature suggests that marital status is associated with hukou conversion, labor market outcomes, co-residency, and gender role attitudes (Moors, 2003; Xiang, 2015; Zhang et al., 2008). Thus, we anticipated that *marital status* could be an important confounder for the association between economic stability, family separation and relations, and family attitudes. If a respondent is currently married, divorced, and widowed, then the *marital status* was coded as 1. The variable was coded as 0 otherwise. At the same time, because marital status is measured at the same time as the other outcome variables could condition some of them, we re-estimated all models without marital status to check stability of main results. These findings are included in the appendix. All other potential confounders were measured at the baseline survey in 2000, and therefore were not affected by this concern.

We adjusted for *father's absence* in childhood because parental migration experience may predispose youth toward both education and migration, and because family separation may also affect children’s education in other ways (Shen et al., 2021). In the 2000 baseline survey, parents were asked about the months of residence at home during the last year. Following precedent in the existing literature, parents who lived at home for 6 months or fewer during the last year (i.e., absent for at least 6 months) were defined as being absent (for example, see Graham and Jordan, 2011; Nguyen, 2016; Zhao and Yu, 2016). According to this definition, 19.6% of sample children’s fathers were absent while only 1.7% of children’s mothers were absent in the year 2000. Due to a small number of absent mothers, we only considered fathers’ absence in this analysis. *Fathers’ absence* is a binary variable, assigned a value of 1 if the father is absent and 0 otherwise.

Other control variables include child demographics (*birth year* and *male*), *number of siblings*, *parents’ education,* and *family income per capita in 2000*. *Male* is a binary variable, coded as 1 if the child was reported as a male in the baseline survey.^8^ *Parents’ education* was operationalized as the average level of both parents’ years of education, to reflect overall household educational attainment. We measured family economic resources by *family income* *per capita, which *was captured by total income in 2000 from various sources (e.g., wages, farm and forest production, livestock, farming, and self-employment), divided by family size. Due to the right-skewed distribution of family income per capita, treating family income per capita as linear may overlook the nonlinear effect of extreme values of income. We, therefore, converted family income per capita to the logarithm form (Ermini and Hendry, 2008).

Table 1 shows descriptive statistics for the sample, by sex. About 21% of young men and young women have non-rural hukou. About 77% of young women are currently employed, while 95% of young men are currently employed. With regard to having a labor contract, less than 50% of young men and young women sign labor contracts with their employers, which implies that more than half of young men and young women have limited access to job-related benefits. Among three types of job-related benefits, the majority of young men and young women enjoy medical insurance, but less than 20% of them had been provided with housing provident funds by their employers. For *geographic separation*, around 37% of young men and young women currently live outside of their home county, showing that most young men and young women are not geographically far away from their parents.

**Table 1.** Descriptive statistics, by sex.

	Female			Male		
	N	Mean/Proportion	SD	N	Mean/Proportion	SD
Economic stability						
Non-rural *hukou*	724	0.21	0.41	853	0.21	0.40
Current employment status	698	0.77	0.42	820	0.95	0.22
Labor contract	672	0.42	0.49	783	0.47	0.50
Medical insurance	735	0.86	0.35	860	0.88	0.32
Old age pension	721	0.46	0.50	858	0.54	0.50
Housing provident fund	720	0.12	0.33	841	0.18	0.39
Geographic separation						
Outside the county	601	0.37	0.48	782	0.36	0.48
Material exchange						
Live at home	261	33.90		489	54.33	
Separate and giving money to parents only	213	27.66		191	21.22	
Separate and receiving money from parents only	47	6.10		48	5.33	
Separate and both giving and receiving money	80	10.39		47	5.22	
Separate and no material exchange	169	21.95		125	13.89	
Intergenerational attitudes						
Ideal to live separately	448	3.04	0.97	634	2.78	0.99
Strongly disagree	20	4.46		19	3.00	
Disagree	125	27.90		130	20.50	
Average	134	29.91		172	27.13	
Agree	153	34.15		267	42.11	
Strongly agree	16	3.57		46	7.26	
Son preference	453	3.19	1.00	638	2.90	1.02
Strongly agree	14	3.09		37	5.80	
Agree	109	24.06		216	33.86	
Average	150	33.11		201	31.50	
Disagree	139	30.68		143	22.41	
Strongly disagree	41	9.05		41	6.43	
Gender role attitudes						
Gendered division of labor	451	3.03	1.06	637	2.60	0.97
Strongly agree	27	5.99		68	10.68	
Agree	140	31.04		262	41.13	
Average	103	22.84		172	27.00	
Disagree	156	34.59		126	19.78	
Strongly disagree	25	5.54		9	1.41	
Gendered childcare responsibilities	451	3.41	0.94	634	3.30	0.97
Strongly agree	9	2.00	19	3.00		
Agree	83	18.40	130	20.50		
Average	107	23.73	172	27.13		
Disagree	218	48.34	267	42.11		
Strongly disagree	34	7.54	46	7.26		
Analytic variable						
Education						
Middle school and less	340	45.82		339	38.92	
Secondary education	306	41.24		404	46.38	
College and above	96	12.94		128	14.7	
Control variables						
Ever married	667	0.86	0.35	844	0.85	0.36
Father’s absence	928	0.19	0.39	1072	0.20	0.40
Birth year	928	1988.91	1.15	1072	1988.91	1.17
Male	928	0.00	0.00	1072	1.00	0.00
Number of siblings	928	2.35	0.78	1072	2.16	0.70
Parents’ education	913	4.95	3.45	1061	5.40	3.35
Family income per capita, 2000	928	1.55	3.63	1072	1.72	2.18

With regard to material exchange, about 34% of young women and 54% of young men lived at home. This gap shows that sons are more likely to stay home with parents. Approximately 44% of young men and 32% of young women have unidirectional or bidirectional material exchanges with their parents. Surprisingly, a non-trivial proportion of young men and young women do not send money home or receive money from home—especially young women. Finally, turning to intergenerational and gender role attitudes, it is evident that young women in the sample tend to be more non-traditional than young men, in terms of intergenerational attitudes. Young women also support more egalitarian gender role attitudes than do young men.

### Analytic approach

We first used binary logistic regression to assess whether and how educational attainment is related to economic stability and living apart from family after adjusting for potential confounders. We then applied multinomial logistic regression to test how different education levels are associated with material exchange, again controlling for potential confounders. Finally, we adopted ordered logit models whether intergenerational and gender role attitudes differ across levels of education, following confounder adjustment. To consider possible gender differences, we conducted the analyses separately for young men and young women. All analyses controlled for child demographic characteristics, marital status, sibship size, father's absence, parents’ education, and logged family income per capita, as well as dummy indicators for townships (i.e., *township dummies*), to adjust for regional and socioeconomic variations.

## Results

We presented estimates from a series of logit models of economic stability outcomes in young adulthood, for men and women (Table 2). Each model includes educational attainment in 2015 and adjusts for a series of childhood socioeconomic and demographic factors and for ever-married status (see corresponding alternative models in Appendix Tables A1-A6).^9^ For both young women and young men, educational attainment is strongly associated with securing an urban residence permit, with having a labor contract, and with access to the housing provident fund. Education is also associated with an increased access to old age pensions for men and women, though the secondary education coefficient is not significant for women and the tertiary coefficient is only marginally significant for men.

**Table 2.** Logit regression models of economic stability outcomes, by sex.

	Non-rural Hukou		Currently employed		Labor contract		Medical insurance		Old age pension		Housing provident fund	
	Female	Male	Female	Male	Female	Male	Female	Male	Female	Male	Female	Male
	Logit	Logit	Logit	Logit	Logit	Logit	Logit	Logit	Logit	Logit	Logit	Logit
	(1)	(2)	(3)	(4)	(5)	(6)	(7)	(8)	(9)	(10)	(11)	(12)
Education (middle school and less)												
Secondary education	1.589***	1.066***	0.497+	0.487	1.247***	1.112***	0.059	0.454	−0.003	0.559*	2.105***	1.727***
	(0.347)	(0.290)	(0.278)	(0.411)	(0.267)	(0.260)	(0.333)	(0.371)	(0.256)	(0.257)	(0.525)	(0.476)
College and above	3.135***	2.787***	1.885**	0.065	2.393***	1.889***	−0.124	1.456+	0.794*	0.759+	3.628***	3.274***
	(0.440)	(0.349)	(0.593)	(0.650)	(0.429)	(0.428)	(0.482)	(0.810)	(0.379)	(0.405)	(0.610)	(0.560)
Ever married	0.662+	−0.466	−3.334**	1.308*	−0.175	0.045	−0.019	−0.582	−0.276	0.498	−1.011*	0.675
	(0.383)	(0.299)	(1.041)	(0.542)	(0.355)	(0.375)	(0.453)	(0.612)	(0.338)	(0.371)	(0.449)	(0.543)
Father’s absence	−0.225	−0.056	0.111	−0.304	−0.013	−0.013	0.185	−0.092	0.203	0.237	−0.236	−0.402
	(0.366)	(0.301)	(0.353)	(0.499)	(0.323)	(0.303)	(0.411)	(0.434)	(0.303)	(0.304)	(0.471)	(0.433)
Birth year	−0.287*	−0.090	−0.025	0.200	0.066	−0.003	−0.073	0.044	−0.204*	−0.021	−0.244	0.054
	(0.113)	(0.100)	(0.113)	(0.180)	(0.106)	(0.104)	(0.131)	(0.154)	(0.101)	(0.103)	(0.154)	(0.146)
Number of siblings	−0.269	0.046	0.141	0.140	0.248	−0.219	−0.026	0.056	−0.000	0.026	−0.059	−0.016
	(0.183)	(0.171)	(0.175)	(0.294)	(0.164)	(0.174)	(0.196)	(0.231)	(0.158)	(0.167)	(0.240)	(0.244)
Parents’ education	0.022	0.080*	−0.028	−0.023	0.015	0.066+	0.030	0.062	0.086*	−0.014	0.103+	0.059
	(0.042)	(0.038)	(0.042)	(0.059)	(0.038)	(0.036)	(0.048)	(0.052)	(0.037)	(0.036)	(0.053)	(0.051)
Family income, 2000	0.040	−0.067	0.134	0.070	0.000	0.033	0.058	0.141	−0.026	−0.053	0.083	−0.050
	(0.036)	(0.069)	(0.136)	(0.107)	(0.036)	(0.049)	(0.090)	(0.125)	(0.027)	(0.048)	(0.055)	(0.079)
Constant	565.434*	174.702	90.470	−587.252+	−103.241	165.879	445.681+	307.666	532.294**	214.243	455.912	196.739
	(224.185)	(199.747)	(205.614)	(335.297)	(241.186)	(182.588)	(261.299)	(249.102)	(205.567)	(156.580)	(301.650)	(216.500)
Township dummies	Yes	Yes	Yes	Yes	Yes	Yes	Yes	Yes	Yes	Yes	Yes	Yes
Observations	584	759	435	381	435	381	411	381	435	381	425	381

*Note:* ^+^*p*< 0.1^ *^p<0.05^ **^p<0.01 ^***^p<0.001; standard errors in parentheses.

For women but not for men, education is associated with employment. However, the lack of an educational effect for young men emerges because the vast majority of young men report being employed, regardless of their education, while there is more variability in young women’s employment status. Part of the story seems to be that for young rural women, marriage is associated with a drastically reduced likelihood of employment, while for men, marriage is associated with higher rates of employment. Finally, education is not consistently associated with medical insurance access, but there is a marginally significant positive coefficient for health insurance for men for tertiary education. Thus, for most of our outcomes, greater education is tied to indicators of economic stability in young adulthood for young men and women.

Table 3 turns to the question of how educational attainment is linked to family separation. Here, a stark gender difference emerges, with education being more tightly tied to intergenerational separation for young men than for young women. The first set of results represents the geographic separation. For young men, both secondary and tertiary education are significantly associated with greater odds of living outside of the county, but there is no significant association for young women. The second set of estimations shows results from multinomial logit models of living arrangements estimated separately for young men and young women. The base category is co-residence with parents. Paralleling the results for geographic separation, findings here suggest a clear gender difference. While there is a strong association of more education with greater residence away from parents for young men, the corresponding results for young women are not statistically significant. For example, for rural young men, the odds of not co-residing with parents among the college-educated are about four times^10^ those of young men with middle school or less education.

**Table 3.** Models of geographic separation (logit) and living arrangements (multinomial logit), by sex.

	Geographic separation		Living arrangements (always co-resides with parents as reference)			
	Outside the county		Does not co-reside with parents	Sometimes co-resides with parents	Does not co-reside with parents	Sometimes co-resides with parents
	Female	Male	Female		Male	
	Logit	Logit	Multinomial logit		Multinomial logit	
	(1)	(2)	(3)	(4)	(5)	(6)
Education (middle school and less)						
Secondary education	0.107	0.725***	−0.520	0.261	0.935***	0.449+
	(0.259)	(0.215)	(0.363)	(0.379)	(0.247)	(0.236)
College and above	0.498	0.829**	−0.633	0.327	1.388***	1.094**
	(0.360)	(0.316)	(0.539)	(0.553)	(0.393)	(0.392)
Ever married	−1.313***	−1.630***	−1.074+	−0.288	−1.232***	−0.055
	(0.360)	(0.286)	(0.562)	(0.608)	(0.350)	(0.375)
Father’s absence	−0.045	−0.112	−0.263	−0.378	−0.621*	−0.484+
	(0.334)	(0.256)	(0.509)	(0.521)	(0.294)	(0.285)
Birth year	−0.109	−0.137	−0.277+	−0.068	−0.107	0.051
	(0.102)	(0.086)	(0.142)	(0.148)	(0.100)	(0.099)
Number of siblings	0.062	−0.037	0.101	−0.020	−0.105	−0.227
	(0.164)	(0.141)	(0.219)	(0.228)	(0.162)	(0.158)
Parents’ education	−0.055	−0.100**	−0.043	−0.062	−0.095**	−0.049
	(0.037)	(0.031)	(0.054)	(0.057)	(0.035)	(0.035)
Family income, 2000	−0.017	0.026	0.209	0.225	0.029	−0.019
	(0.073)	(0.046)	(0.183)	(0.183)	(0.056)	(0.058)
Constant	217.180	272.931	554.125+	135.621	213.902	−102.323
	(202.451)	(170.162)	(283.575)	(293.740)	(199.523)	(196.335)
Township dummies	Yes	Yes	Yes	Yes	Yes	Yes
Observations	464	655	482	482	685	685

*Note:* ^+^*p*< 0.1^ *^p<0.05^ **^p<0.01 ^***^p<0.001; standard errors in parentheses.

Using multinomial logistic regression, we analyzed the association between education and intergenerational exchange (Table 4). The reference category here was “co-residency”, compared to separate living with varying exchange types: giving money to parents only, receiving money from parents only, reciprocal exchange, and no material exchange. Table 4, similar to Table 3, indicates significant gender differences. For women, a higher level of education correlates with a reduced likelihood of two outcomes compared to co-residence: living away while providing financial support to parents, and living apart with a complete cessation of exchange. Education has no significant association with other exchange patterns. In contrast, for men, our results in Table 4 demonstrate that education is consistently associated with all forms of separate living with material exchange, relative to co-residence. However, men's education does not link to the likelihood of completely halting financial exchange if living apart. This divergent finding aligns with the interpretation that is associated with trading material exchange for family togetherness among men but not women.

**Table 4.** Multinomial logit models of material exchange, by sex.

	Material exchange (co-resident as reference)				Material exchange (co-resident as reference)			
	Separate and giving money to parents only	Separate and receiving money from parents only	Separate and both giving and receiving money	Separate and no material exchange	Separate and giving money to parents only	Separate and receiving money from parents only	Separate and both giving and receiving money	Separate and no material exchange
	Female				Male			
	Multinomial logit				Multinomial logit			
	(1)	(2)	(3)	(4)	(5)	(6)	(7)	(8)
Education (middle school and less)								
Secondary education	−0.616*	0.092	−0.197	−0.918**	0.653**	2.341***	0.901+	0.025
	(0.269)	(0.497)	(0.399)	(0.301)	(0.236)	(0.653)	(0.490)	(0.280)
College and above	−1.011*	−0.148	−0.032	−1.547**	0.767*	2.811***	1.920**	−0.005
	(0.401)	(0.679)	(0.498)	(0.485)	(0.320)	(0.714)	(0.596)	(0.403)
Ever married	−0.712*	−0.944	−1.069*	−0.834*	−0.993***	−2.286***	−0.277	−1.452***
	(0.357)	(0.574)	(0.490)	(0.412)	(0.299)	(0.482)	(0.577)	(0.319)
Father’s absence	−0.169	0.721	−0.632	0.112	−0.222	−0.510	0.178	−0.269
	(0.351)	(0.577)	(0.537)	(0.380)	(0.270)	(0.565)	(0.481)	(0.336)
Birth year	−0.298**	−0.101	−0.071	−0.272*	−0.114	−0.241	−0.113	−0.157
	(0.106)	(0.181)	(0.147)	(0.118)	(0.090)	(0.174)	(0.170)	(0.109)
Number of siblings	−0.040	0.075	0.541*	0.111	0.052	0.006	−0.595+	0.173
	(0.174)	(0.304)	(0.235)	(0.192)	(0.151)	(0.300)	(0.332)	(0.186)
Parents’ education	−0.029	0.013	0.058	−0.010	−0.070*	0.012	−0.170**	−0.055
	(0.039)	(0.063)	(0.057)	(0.043)	(0.034)	(0.062)	(0.063)	(0.040)
Family income, 2000	0.016	−0.070	0.081	−0.008	0.024	−0.127	0.161+	0.037
	(0.074)	(0.161)	(0.067)	(0.037)	(0.052)	(0.134)	(0.091)	(0.063)
Constant	593.793**	200.951	141.447	541.507*	226.195	476.753	206.805	312.428
	(211.497)	(360.447)	(291.810)	(234.166)	(179.809)	(346.334)	(2898.199)	(215.861)
Township dummies	Yes	Yes	Yes	Yes	Yes	Yes	Yes	Yes
Observations	613	613	613	613	794	794	794	794

*Note:* ^+^*p*< 0.1^ *^p<0.05^ **^p<0.01 ^***^p<0.001; standard errors in parentheses.

We tested hypotheses regarding family and gender role attitudes with ordered logistic regression models (Tables 5 and 6). Table 5 shows that for men and women, a higher level of education (i.e., tertiary education and sometimes secondary education) is associated with espousing more non-traditional co-residence preferences (living separately) and having less son preference. Moreover, Table 6 shows that more educated young men and women experienced greater odds of espousing egalitarian norms about the gender division of labor and responsibilities for childcare.

**Table 5.** Ordered logit models of intergenerational attitudes, by sex.

	Ideal to live separately		Less son preference	
	Female	Male	Female	Male
	Ordered logit	Ordered logit	Ordered logit	Ordered logit
	(1)	(2)	(5)	(6)
Education (middle school and less)				
Secondary education	0.358	0.386*	0.527*	0.252
	(0.221)	(0.184)	(0.216)	(0.182)
College and above	1.230***	0.610*	0.756*	0.504*
	(0.317)	(0.244)	(0.309)	(0.240)
Ever married	0.482	−0.432	−0.927	−0.242
	(0.817)	(0.840)	(0.957)	(0.825)
Father’s absence	−0.660*	−0.066	0.027	0.084
	(0.301)	(0.210)	(0.297)	(0.207)
Birth year	−0.037	−0.095	−0.001	0.046
	(0.088)	(0.071)	(0.085)	(0.070)
Number of siblings	0.253+	−0.061	0.006	−0.207+
	(0.136)	(0.119)	(0.134)	(0.116)
Parents’ education	0.021	−0.042	−0.009	−0.032
	(0.032)	(0.026)	(0.031)	(0.026)
Family income, 2000	0.009	0.024	0.008	0.011
	(0.022)	(0.038)	(0.021)	(0.037)
/cut1	−75.973	−193.544	−6.216	87.352
	(174.472)	(141.934)	(168.733)	(140.026)
/cut2	−73.347	−191.191	−3.686	89.851
	(174.472)	(141.930)	(168.735)	(140.028)
/cut3	−71.852	−189.891	−2.171	91.263
	(174.472)	(141.927)	(168.734)	(140.028)
/cut4	−68.745	−187.081	−0.137	93.128
	(174.466)	(141.924)	(168.734)	(140.029)
Township dummies	Yes	Yes	Yes	Yes
Observations	439	623	439	623

*Note:* ^+^*p*< 0.1^ *^p<0.05^ **^p<0.01 ^***^p<0.001; standard errors in parentheses.

**Table 6.** Ordered logit models of gender role attitudes, by sex.

	Less gendered division of labor		Less gendered childcare responsibilities	
	Female	Male	Female	Male
	Ordered logit	Ordered logit	Ordered logit	Ordered logit
	(1)	(2)	(3)	(4)
Education (middle school and less)				
Secondary education	0.653**	0.448*	0.452*	0.020
	(0.219)	(0.186)	(0.224)	(0.183)
College and above	1.454***	0.668**	0.685*	0.648*
	(0.318)	(0.247)	(0.314)	(0.254)
Ever married	0.139	−0.776	−0.095	−0.342
	(0.887)	(0.864)	(0.877)	(0.849)
Father’s absence	0.308	−0.032	0.508+	0.304
	(0.295)	(0.209)	(0.303)	(0.206)
Birth year	−0.042	−0.051	0.059	0.061
	(0.086)	(0.071)	(0.088)	(0.072)
Number of siblings	0.099	−0.076	0.110	0.047
	(0.136)	(0.119)	(0.137)	(0.120)
Parents’ education	0.067*	−0.016	−0.001	0.057*
	(0.032)	(0.026)	(0.032)	(0.026)
Family income, 2000	−0.012	0.021	−0.023	0.032
	(0.020)	(0.039)	(0.021)	(0.044)
/cut1	−86.896	−104.146	113.277	116.726
	(171.658)	(141.004)	(175.769)	(144.020)
/cut2	−84.491	−101.769	116.026	119.063
	(171.660)	(141.002)	(175.776)	(144.024)
/cut3	−83.376	−100.432	117.272	120.375
	(171.659)	(141.000)	(175.779)	(144.025)
/cut4	−80.645	−97.320	120.304	123.152
	(171.652)	(140.998)	(175.780)	(144.027)
Township dummies	Yes	Yes	Yes	Yes
Observations	442	620	442	620

*Note:* ^+^*p*< 0.1^ *^p<0.05^ **^p<0.01 ^***^p<0.001; standard errors in parentheses.

## Discussion and conclusions

Economic disparities between urban and rural areas drive rural youth in China to migrate for better opportunities, often distancesing them from familial networks. Education may play a key role in this process. In this paper, we considered whether educational advancement is associated with economic security for rural youth in young adulthood, and analyzed whether it is also linked to separation from natal families or transformations in rural family relationships, as might be expected under second demographic transition theory. Focusing on young adults from rural northwest China, we estimated relationships between secondary and tertiary educational attainment and (1) economic stability, (2) geographic separation and material exchange, and (3) traditional family and gender role attitudes, after adjusting for many potential confounders.

We did find some evidence that higher educational attainment is associated with greater family separation and more material exchange, at least for young men. Research elsewhere has suggested that remittances and communications technology can offer alternative ways for generations to support each other in terms of material exchange, which can also be an important component of intergenerational relationships (Zimmer and Kwong, 2003). Though certain aspects of intergenerational support cannot be easily accomplished without proximity, the rise and widespread adoption of mobile communication and banking technologies in China have fundamentally changed the possibilities for connection and support across distance in the years between the waves of the study here. Distance may be much less of a strain on intergenerational relationships than in the past, due to communication technology, but distance certainly implies a change in the mode of communication and support.

Moreover, the gender differences noted here are significant. Young women with more education are more likely to be employed, have better jobs, and are no more disconnected from families than otherwise might be the case. For young men, the story is more nuanced. Those who are more educated do not enjoy an employment advantage, as most of the cohort are employed. However, they do enjoy better benefits such as having labor contracts, pensions, and housing funds than their less-educated counterparts. Additionally, compared to less-educated young men, educated young men are more likely to be away from home. For good or ill, these men are also more likely to swap material exchange for co-residence and direct intergenerational support. Overall, the accessibility to educational opportunities in rural communities thus appears to be associated with both economic benefits and some transformations for rural youth and their natal families, but the transformations may be different, and possibly more pronounced, for the natal families of educated young men than for those of educated young women.^11^

This study is limited in several ways. First, our study relies on a sample based on one province in China, which restricts generalizability, and the sample does not contain sufficient numbers of children from recognized ethnic minority groups to permit any analysis of ethnic differences in observed patterns. In addition, most measures of the dependent and independent variables in the data were collected through either self-reports or proxy reports by parents and are hence subject to reporting errors. Finally, the 2015 survey data may not reflect the most recent internal migration trends in China. In 2014, a major hukou reform was passed aiming to eliminate distinctions between rural and urban status by replacing the system with a residential permit. The association between hukou status and youth education could shift once this reform is fully implemented nationwide.

Despite these limitations, we believe that this case study offers a valuable contribution by representing a demographically substantial population of rural youth. Moreover, by utilizing a purpose-designed 15-year longitudinal dataset, we are able to examine the interplay between rural childhood circumstances and subsequent outcomes in adulthood (Shen et al., 2021). The key insight of interest to demographic theories of family change is that educational advancement is associated with more urban-based residence and employment and thus reinforces processes set to disrupt traditional family relations—patterns of family co-residence, forms of intergenerational exchange, and attitudes toward gender and childbearing. It is significant that many of these pressures on family relationships tend to be more striking for young rural men than for young women, who were already expected in earlier periods to leave natal homes upon marriage. Erosion of established forms of family interaction for young rural men may compound the isolation of this group, who are at risk of many forms of social and economic exclusion in contemporary Chinese society.

## Notes

1. In line with the findings of low returns to schooling in rural areas, on field-based study raised questions about the efficacy of the education system in providing real pathways to economic opportunity for youth in some of China’s poorest rural communities (Wu, 2016).

2. Higher education among women has been reliably associated in past decades with delayed marriage and childbearing, lower fertility rates, and a greater acceptance and preference for cohabitation outside of marriage (e.g., Jejeebhoy, 1995; Skirbekk, 2008), though the association of education with delayed marriage sometimes reverses over time as fertility drops, as it has in China for women (Yu and Xie, 2015).

3. While it is not our focus in this paper, urban areas generally have a higher concentration of resources, the transition from rural to urban education can also be a daunting experience for rural children. Impediments such as inadequate housing, discrimination, and limited access to quality healthcare pose substantial challenges for these children. A study by Logan and Alba (1993) found that rural children in urban areas are more likely to live in overcrowded conditions, which negatively impacts their academic performance and physical health.

4. In addition, the revolutionary socialist agenda in China carried certain elements of a gender-egalitarian ideology, and more recently, global feminist thought has become more influential in China. Notions of economic independence as well as individualism have become influential and have entered into discussions of gender dynamics (Liu et al., 2013). Education could bring more exposure to these views.

5. One reviewer suggested that in Australia and the UK, rural youth who have labor contracts still experience job precarity (Chesters and Cuervo, 2019; Thomson et al., 2004). However, in China, rural-urban migrant workers tend to be uncontracted or have a short-term contract which limits their access to social insurance (Gao et al., 2012). The implementation of the “China Labor Contract Law” in 2008 aimed at enhancing the protection of labor rights for migrant and temporary workers by encouraging employers to sign long-term contracts with workers. Prior research suggested that having a labor contract significantly improves the accessibility of social insurance and promotes urban integration of migrant workers (Gao et al., 2012; Zhao and Tang, 2022).

6. The housing provident fund was first introduced in Shanghai in 1991 to support affordable housing for ordinary households. Employees are required to contribute a portion of salaries to this fund and employers contribute a similar amount of money to the employees’ accounts under this scheme. Employees are allowed to withdraw this fund for purchasing, constructing, or remodeling own houses (Yeung and Howes, 2006). Housing provident funds along with pensions, medical, unemployment, work injury, and maternity insurance constitute the primary employment-based social benefits in China (Gao et al., 2012).

7. As a robustness check, we repeated the analysis using original categories, and the gender and educational patterns were not subject to change.

8. We only included a binary specification for gender.

9. Because the decision to marry is related to other young adult outcomes, we also estimated all models with and without adjusting for ever-married status. Models without ever-married status are included in Appendix 1.

10. Exponentiating 1.388 yields an odds ratio of 4.

11. The traditional Chinese family is patrilineal and patrilocal, daughters are not viewed as members of the descent line and are expected to join husbands’ family and take care of parents-in-law (Greenhalgh, 1985). Under this tradition, married daughters are constrained to provide financial support to their natal family. However, (Xie and Zhu, 2009) showed that this traditional pattern has reversed in urban China, where married daughters give more money to parents than married sons.

## Appendix 1 Full set of tables without the ever married variable.

**Appendix Table A1.** Descriptive statistics by sex (alternative table without *ever married*).

	Female			Male		
	N	Mean/Proportion	SD	N	Mean/Proportion	SD
Economic stability						
Non-rural *hukou*	724	0.21	0.41	853	0.21	0.40
Current employment status	698	0.77	0.42	820	0.95	0.22
Labor contract	672	0.42	0.49	783	0.47	0.50
Medical insurance	735	0.86	0.35	860	0.88	0.32
Old age pension	721	0.46	0.50	858	0.54	0.50
Housing provident fund	720	0.12	0.33	841	0.18	0.39
Geographic separation						
Outside the county	601	0.37	0.48	782	0.36	0.48
Material exchange						
Live at home	261	33.90		489	54.33	
Separate and giving money to parents only	213	27.66		191	21.22	
Separate and receiving money from parents only	47	6.10		48	5.33	
Separate and both giving and receiving money	80	10.39		47	5.22	
Separate and no material exchange	169	21.95		125	13.89	
Intergenerational attitudes						
Ideal to live separately	448	3.04	0.97	634	2.78	0.99
Strongly disagree	20	4.46		19	3.00	
Disagree	125	27.90		130	20.50	
Average	134	29.91		172	27.13	
Agree	153	34.15		267	42.11	
Strongly agree	16	3.57		46	7.26	
Son preference	453	3.19	1.00	638	2.90	1.02
Strongly agree	14	3.09		37	5.80	
Agree	109	24.06		216	33.86	
Average	150	33.11		201	31.50	
Disagree	139	30.68		143	22.41	
Strongly disagree	41	9.05		41	6.43	
Gender role attitudes						
Gendered division of labor	451	3.03	1.06	637	2.60	0.97
Strongly agree	27	5.99		68	10.68	
Agree	140	31.04		262	41.13	
Average	103	22.84		172	27.00	
Disagree	156	34.59		126	19.78	
Strongly disagree	25	5.54		9	1.41	
Gendered childcare responsibilities	451	3.41	0.94	634	3.30	0.97
Strongly agree	9	2.00	19	3.00		
Agree	83	18.40	130	20.50		
Average	107	23.73	172	27.13		
Disagree	218	48.34	267	42.11		
Strongly disagree	34	7.54	46	7.26		
Analytic variable						
Education						
Middle school and less	340	45.82		339	38.92	
Secondary education	306	41.24		404	46.38	
College and above	96	12.94		128	14.7	
Control variables						
Father’s absence	928	0.19	0.39	1072	0.20	0.40
Birth year	928	1988.91	1.15	1072	1988.91	1.17
Male	928	0.00	0.00	1072	1.00	0.00
Number of siblings	928	2.35	0.78	1072	2.16	0.70
Parents’ education	913	4.95	3.45	1061	5.40	3.35
Family income per capita, 2000	928	1.55	3.63	1072	1.72	2.18

**Appendix Table A2.** Logit models of economic stability outcomes, by sex (alternative specification without *ever married*).

	Non-rural Hukou		Currently employed		Labor contract		Medical insurance		Old age pension		Housing provident fund	
	Female	Male	Female	Male	Female	Male	Female	Male	Female	Male	Female	Male
	Logit	Logit	Logit	Logit	Logit	Logit	Logit	Logit	Logit	Logit	Logit	Logit
	(1)	(2)	(3)	(4)	(5)	(6)	(7)	(8)	(9)	(10)	(11)	(12)
Education (middle school and less)												
Secondary education	1.510***	1.086***	0.538*	0.506	1.251***	1.111***	0.060	0.475	0.009	0.559*	2.132***	1.726***
	(0.341)	(0.290)	(0.271)	(0.407)	(0.267)	(0.260)	(0.333)	(0.370)	(0.255)	(0.256)	(0.520)	(0.475)
College and above	3.053***	2.785***	1.838**	0.088	2.392***	1.889***	−0.124	1.464+	0.797*	0.750+	3.589***	3.260***
	(0.435)	(0.349)	(0.582)	(0.640)	(0.427)	(0.428)	(0.482)	(0.807)	(0.378)	(0.404)	(0.599)	(0.559)
Father’s absence	−0.234	−0.071	0.120	−0.203	−0.010	−0.011	0.186	−0.138	0.207	0.247	−0.274	−0.400
	(0.365)	(0.301)	(0.339)	(0.500)	(0.323)	(0.303)	(0.411)	(0.428)	(0.303)	(0.304)	(0.470)	(0.432)
Birth year	−0.284*	−0.071	0.013	0.143	0.068	−0.004	−0.073	0.055	−0.200*	−0.031	−0.237	0.038
	(0.113)	(0.100)	(0.111)	(0.174)	(0.106)	(0.104)	(0.131)	(0.153)	(0.101)	(0.103)	(0.151)	(0.145)
Number of siblings	−0.280	0.058	0.153	0.102	0.251	−0.220	−0.025	0.079	0.006	0.013	−0.038	−0.046
	(0.183)	(0.170)	(0.169)	(0.286)	(0.164)	(0.174)	(0.196)	(0.230)	(0.157)	(0.165)	(0.236)	(0.243)
Parents’ education	0.018	0.083*	−0.007	−0.028	0.017	0.065+	0.030	0.062	0.088*	−0.016	0.110*	0.056
	(0.042)	(0.038)	(0.040)	(0.058)	(0.038)	(0.036)	(0.047)	(0.052)	(0.037)	(0.036)	(0.053)	(0.050)
Family income, 2000	0.042	−0.065	0.103	0.057	−0.000	0.033	0.058	0.138	−0.027	−0.052	0.080	−0.047
	(0.036)	(0.069)	(0.127)	(0.104)	(0.036)	(0.049)	(0.090)	(0.124)	(0.027)	(0.048)	(0.055)	(0.079)
Township dummies	Y	Y	Y	Y	Y	Y	Y	Y	Y	Y	Y	Y
Constant	560.228*	136.854	−25.690	−281.128	−137.346	6.293	146.894	−108.405	397.367*	61.142	466.224	−79.413
	(224.284)	(198.757)	(221.809)	(346.605)	(211.556)	(206.788)	(260.365)	(305.071)	(201.253)	(204.010)	(300.328)	(287.975)
Observations	584	759	435	381	435	381	411	381	435	381	425	381

*Note:* ^+^*p*< 0.1^ *^*p*<0.05^ **^*p*<0.01 ^***^*p*<0.001; standard errors in parentheses.

**Appendix Table A3.** Models of geographic separation (logit) and living arrangements (multinomial logit), by sex (alternative specification without *ever married*).

	Geographic separation		Living arrangements (always co-resides with parents as reference)			
	Outside the county		Does not co-reside with parents	Sometimes co-resides with parents	Does not co-reside with parents	Sometimes co-resides with parents
	Female	Male	Female		Male	
	Logit	Logit	Multinomial logit		Multinomial logit	
	(1)	(2)	(3)	(4)	(5)	(6)
Education (middle school and less)						
Secondary education	0.190	0.683**	−0.423	0.285	0.919***	0.432+
	(0.254)	(0.209)	(0.357)	(0.374)	(0.244)	(0.235)
College and above	0.585+	0.856**	−0.545	0.332	1.407***	1.067**
	(0.352)	(0.306)	(0.530)	(0.546)	(0.388)	(0.389)
Father’s absence	−0.042	−0.061	−0.263	−0.369	−0.578*	−0.467
	(0.327)	(0.249)	(0.507)	(0.521)	(0.291)	(0.284)
Birth year	−0.081	−0.059	−0.267+	−0.070	−0.053	0.053
	(0.100)	(0.082)	(0.142)	(0.148)	(0.098)	(0.097)
Number of siblings	0.056	−0.068	0.119	−0.008	−0.139	−0.235
	(0.161)	(0.138)	(0.218)	(0.228)	(0.160)	(0.158)
Parents’ education	−0.045	−0.092**	−0.035	−0.060	−0.090*	−0.048
	(0.036)	(0.030)	(0.054)	(0.056)	(0.035)	(0.035)
Family income, 2000	−0.023	0.025	0.198	0.216	0.032	−0.018
	(0.072)	(0.045)	(0.181)	(0.181)	(0.056)	(0.058)
Constant	161.003	116.254	532.772+	139.990	104.904	−106.164
	(199.289)	(162.536)	(282.260)	(294.282)	(194.755)	(193.912)
Township dummies	Yes	Yes	Yes	Yes	Yes	Yes
Observations	464	655	482	482	685	685

*Note:* ^+^*p*< 0.1^ *^*p*<0.05^ **^*p*<0.01 ^***^*p*<0.001; standard errors in parentheses.

**Appendix Table A4.** Multinomial logit models of material exchange, by sex (alternative specification without *ever married*).

	Material exchange (co-resident as reference)				Material exchange (co-resident as reference)			
	Separate and giving money to parents only	Separate and receiving money from parents only	Separate and both giving and receiving money	Separate and no material exchange	Separate and giving money to parents only	Separate and receiving money from parents only	Separate and both giving and receiving money	Separate and no material exchange
	Female				Male			
	Multinomial logit				Multinomial logit			
	(1)	(2)	(3)	(4)	(5)	(6)	(7)	(8)
Education (middle school and less)								
Secondary education	−0.556*	0.162	−0.087	−0.867**	0.665**	2.375***	0.855+	0.033
	(0.267)	(0.495)	(0.395)	(0.298)	(0.234)	(0.648)	(0.490)	(0.276)
College and above	−0.942*	−0.117	0.061	−1.494**	0.791*	2.921***	1.967***	−0.006
	(0.396)	(0.678)	(0.495)	(0.482)	(0.317)	(0.702)	(0.593)	(0.398)
Father’s absence	−0.182	0.695	−0.651	0.110	−0.220	−0.487	0.148	−0.259
	(0.350)	(0.574)	(0.534)	(0.378)	(0.268)	(0.541)	(0.480)	(0.330)
Birth year	−0.294**	−0.094	−0.071	−0.266*	−0.077	−0.052	−0.098	−0.098
	(0.106)	(0.180)	(0.146)	(0.117)	(0.088)	(0.163)	(0.168)	(0.105)
Number of siblings	−0.026	0.091	0.554*	0.124	0.041	−0.067	−0.604+	0.142
	(0.173)	(0.303)	(0.235)	(0.191)	(0.149)	(0.293)	(0.330)	(0.181)
Parents’ education	−0.025	0.017	0.062	−0.004	−0.068*	0.006	−0.172**	−0.051
	(0.039)	(0.063)	(0.057)	(0.042)	(0.034)	(0.061)	(0.063)	(0.040)
Family income, 2000	0.014	−0.072	0.076	−0.010	0.029	−0.095	0.173+	0.053
	(0.074)	(0.162)	(0.067)	(0.037)	(0.051)	(0.124)	(0.091)	(0.061)
Constant	584.198**	186.315	139.609	529.720*	152.997	100.068	176.432	193.414
	(210.516)	(357.544)	(289.794)	(232.899)	(176.015)	(324.455)	(4138.602)	(208.865)
Township dummies	Y	Y	Y	Y	Y	Y	Y	Y
Observations	613	613	613	613	794	794	794	794

*Note:* ^+^*p*< 0.1^ *^*p*<0.05^ **^*p*<0.01 ^***^*p*<0.001; standard errors in parentheses.

**Appendix Table A5.** Ordered logit models of intergenerational attitudes, by sex (alternative specification without *ever married*).

	Ideal to live separately		Less son preference	
	Female	Male	Female	Male
	Ordered logit	Ordered logit	Ordered logit	Ordered logit
	(1)	(2)	(3)	(4)
Education (middle school and less)				
Secondary education	0.352	0.391*	0.528*	0.256
	(0.221)	(0.184)	(0.216)	(0.182)
College and above	1.223***	0.607*	0.760*	0.504*
	(0.317)	(0.244)	(0.309)	(0.240)
Father’s absence	−0.658*	−0.066	0.024	0.082
	(0.301)	(0.210)	(0.297)	(0.206)
Birth year	−0.039	−0.094	0.001	0.046
	(0.088)	(0.071)	(0.085)	(0.070)
Number of siblings	0.243+	−0.062	0.022	−0.207+
	(0.135)	(0.119)	(0.133)	(0.116)
Parents’ education	0.021	−0.042	−0.010	−0.032
	(0.032)	(0.026)	(0.031)	(0.026)
Family income, 2000	0.010	0.023	0.008	0.011
	(0.022)	(0.038)	(0.021)	(0.037)
/cut1	−80.034	−191.012	−0.950	88.114
	(174.283)	(141.845)	(168.643)	(139.988)
/cut2	−77.407	−188.659	1.578	90.613
	(174.282)	(141.841)	(168.644)	(139.991)
/cut3	−75.913	−187.359	3.092	92.025
	(174.282)	(141.838)	(168.644)	(139.991)
/cut4	−72.809	−184.550	5.124	93.891
	(174.276)	(141.835)	(168.644)	(139.991)
Township dummies	Y	Y	Y	Y
Observations	439	623	439	623

*Note:* ^+^*p*< 0.1^ *^*p*<0.05^ **^*p*<0.01 ^***^*p*<0.001; standard errors in parentheses.

**Appendix Table A6.** Ordered logit models of gender role attitudes, by sex (alternative specification without *ever married*).

	Less gendered division of labor		Less gendered childcare responsibilities	
	Female	Male	Female	Male
	Ordered logit	Ordered logit	Ordered logit	Ordered logit
	(1)	(2)	(3)	(4)
Education (middle school and less)				
Secondary education	0.652**	0.455*	0.453*	0.025
	(0.219)	(0.186)	(0.224)	(0.183)
College and above	1.452***	0.660**	0.685*	0.646*
	(0.318)	(0.246)	(0.314)	(0.254)
Father’s absence	0.309	−0.038	0.507+	0.304
	(0.295)	(0.209)	(0.303)	(0.206)
Birth year	−0.043	−0.050	0.059	0.061
	(0.086)	(0.071)	(0.088)	(0.072)
Number of siblings	0.097	−0.076	0.112	0.047
	(0.135)	(0.119)	(0.136)	(0.120)
Parents’ education	0.067*	−0.016	−0.001	0.057*
	(0.032)	(0.026)	(0.032)	(0.026)
Family income, 2000	−0.012	0.020	−0.023	0.031
	(0.020)	(0.039)	(0.021)	(0.044)
/cut1	−88.072	−100.771	114.157	118.620
	(171.484)	(140.913)	(175.598)	(143.930)
/cut2	−85.666	−98.395	116.906	120.957
	(171.486)	(140.911)	(175.606)	(143.934)
/cut3	−84.552	−97.060	118.152	122.269
	(171.484)	(140.910)	(175.609)	(143.935)
/cut4	−81.820	−93.950	121.184	125.046
	(171.477)	(140.908)	(175.610)	(143.937)
Township dummies	Y	Y	Y	Y
Observations	442	620	442	620

*Note:* ^+^*p*< 0.1^ *^*p*<0.05^ **^*p*<0.01 ^***^*p*<0.001; standard errors in parentheses.
